# Preliminary Comparison of Transseptal Microscopic Versus Endoscopic Transsphenoidal Surgery in a University Teaching Hospital

**DOI:** 10.21315/mjms2022.29.1.7

**Published:** 2022-02-23

**Authors:** Zaitun Zakaria, Zamzuri Idris, Jafri Malin Abdullah, Baharudin Abdullah, Abdul Rahman Izaini Ghani

**Affiliations:** 1 Department of Neurosciences, School of Medical Sciences, Universiti Sains Malaysia, Kelantan, Malaysia; 2 Hospital Universiti Sains Malaysia, Universiti Sains Malaysia, Kelantan, Malaysia; 3 Brain and Behaviour Cluster, School of Medical Sciences, Universiti Sains Malaysia, Kelantan, Malaysia; 4 Department of Otorhinolaryngology-Head and Neck Surgery, School of Medical Sciences, Universiti Sains Malaysia, Kelantan, Malaysia

**Keywords:** endoscopic, pituitary, sella, suprasellar, transsphenoidal, transseptal

## Abstract

**Background:**

Transsphenoidal surgery (TSS) is an increasing preferred treatment for sella lesions. In a university teaching hospital, the novel endoscopic TSS was adopted with ongoing resident teaching. We evaluated a single institutional series of preliminary comparisons of transseptal microscopic with endoscopic TSS.

**Methods:**

A retrospective data analysis included 37 patients and 19 patients who underwent microscopic and endoscopic TSS, respectively. The demographic characteristics of the patients, intra-operative analyses, morbidity, mortality and visual assessments were included in this analysis.

**Results:**

The study included 31 men and 25 women, and median age at surgery was 49 years old (range 14–70 years old). There were no differences between the rates of cerebrospinal fluid (CSF) fistula, sinus complications, anterior pituitary hormone deficiency and diabetes insipidus between the groups. Total length of stay and intensive care unit stay were similar between the groups. Patients who underwent endoscopic TSS were at significantly increased risk of epistaxis (*P* = 0.010), respiratory event (*P* = 0.014) and post-operative visual deterioration prior to discharge (*P* = 0.032).

**Conclusion:**

Endoscopic TSS is a promising procedure that allows sufficient visualisation of the surgical field and adequate tumour removal. It is comparable to microscopic TSS but has a higher complication rate notably due to steep learning curve required to gain the expertise.

## Introduction

The minimally invasive extra-cerebral approach to the sellar and suprasellar region is becoming more attractive and has regained much interest among neurosurgeons. In contrast to craniotomy, transnasal transsphenoidal surgery (TSS) uses the body’s own pneumatic cavities to reach a small space with highly relevant anatomical elements (1). The first documented TSS was performed in 1907 by the Austrian neurosurgeon Hermann Schloffer (2, 3). The popularisation of the transsphenoidal route reached two prominent pituitary surgeons: Harvey Cushing from the United States in 1909 and Oskar Hirsch from Vienna, Austria in 1910. Further evolutions and modifications were achieved by Cushing’s apprentice, the Scottish surgeon Norman Dott (4). Three decades later, the relevant knowledge was acquired by Gerard Guiot, who began the transsphenoidal approach in Paris in 1957 (5). In the 1960s, Guiot met a visiting fellow from Montreal, Canada called Jules Hardy, who became much interested in this technique. He brought back the knowledge and continued performing operations using pre-operative encephalography and intra-operative radiofluoroscopy. His frustration at the time was due to the limited visualisation of the tumour, causing incomplete tumour removal. This frustration ultimately became the light of his solution and, in 1965, he became the first surgeon to use an operating microscope, hence microscopic TSS (6, 7).

The endoscopic technique was created in the early 1980s (8). The attraction broadened after the French otolaryngologists, Jankowski and colleagues, presented their series of outcomes in 1990 (9). Since then, many centres have started to become familiar with both approaches or have endeavoured to attempt endoscopic TSS (10, 11). Thus, the relative efficacy rates of microscopic TSS and of endoscopic TSS have been continually compared. At the university teaching hospital, both approaches are available, with endoscopic TSS been newly introduced in 2014. The adoption of endoscopic technique while at the same time training the residents, requires passion and certain amount of time. Hence, we determined to evaluate both techniques used to treat sella and suprasellar lesions, with the aim of objectifying their differences in terms of results and complication rates (1).

## Methods

A retrospective data collection was conducted, where the data of patients who underwent TSS between 2011 and 2020 at the Neurosurgery Department of the Hospital Universiti Sains Malaysia were retrieved. The patient details were identified from operating theatre (OT) books and further confirmed from the hospital’s medical records and laboratory results. To minimise the selection bias and reliability of this study, data abstraction was conducted by a single investigator who had not been involved with the patients’ care during the hospital admission, operation, and clinic follow-up process.

A total of 65 patients were identified and exclusion was utilised for inaccessible medical folders, those lost to follow-up, patients who only had a biopsy and cases of the extended transsphenoidal approach (e.g. for a clival tumour). Fifty-six patients were selected for the present analysis and were divided into two groups: i) the microscopic TSS and ii) endoscopic TSS. At the time when endoscopic TSS became available, the decision was made based on consensus decision with the patient (or family members), and the surgeon’s opinion of the best possible route to tackle the space occupying lesion. The demographic characteristics of the patients, and their intra-operative analyses, morbidity, mortality and visual assessment were included in this analysis. Using magnetic resonance imaging (MRI) of the pituitary protocol, the pre- and post-operative tumour volumes were calculated to ascertain the volume reduction.

Complications were divided into specific and general. Those specific to transsphenoidal surgery include cerebrospinal fluid (CSF) fistula, haemorrhage or epistaxis, sinus complication and medical complications; diabetes insipidus and anterior pituitary hormone deficiency. The mortality rate was calculated as any death that occurred during hospital admission and at follow-up at 1 year (but as a direct consequence of surgical complications). Pre-and post-operatively, patients were subjected to endocrinological and neuro-ophthalmological assessments, including Humphrey visual field testing. Patients were then followed up for at least 1 year and conditions were described as either ‘worse’, ‘no improvement’, ‘somewhat improved’ or ‘resolved’.

### Operative Procedure

The operative procedure for microscopic TSS is solely performed by neurosurgeons, while endoscopic TSS is assisted by the ear, nose and throat (ENT) surgeons (for nasal surgery until the sphenoid phase); these are described as below.

One dose of antibiotic with primarily Gram-positive coverage was given routinely within 60 min of the incision. The patient’s head was pinned with an initial head flexion of 10°–20°, followed by a 10° head turn to the left and a 10°–20° head extension. An oral endotracheal tube was used to administer general anaesthesia and a pharyngeal pack was inserted. After taping the eyes and packing the oral region, the midfacial area was cleaned with povidone iodine and draped in a quadrangular fashion with towels. The local vascular pedicle around the nose was packed with gauze soaked in Moffett’s solution (cocaine 10%, sodium bicarbonate, adrenaline 1:10,000 and sterile saline). The surgeon operated on the right side of the patient, with an assistant positioned on the opposite side.

**Figure 1:**
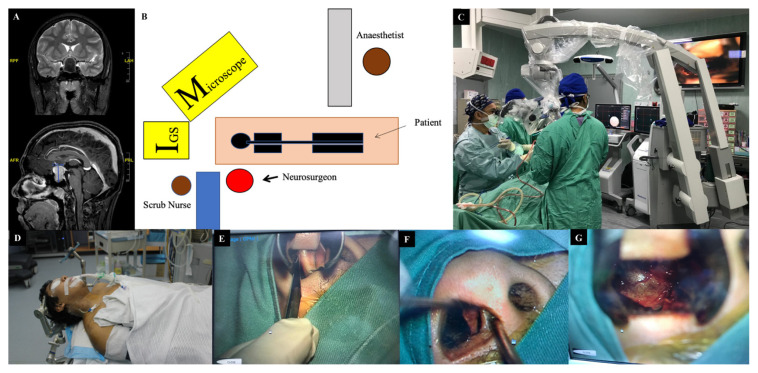
Illustration of microscopic TSS. (A) A T2W (above) and T1W postgadolinium (below) MRI brain shows a sella and suprasellar lesion in a 17-year-old male who presented with a raised intracranial pressure and progressive deterioration of vision. (B–C) Depictions of the OT arrangement, with the main surgeon sitting to the right of the patient. (D) The patient’s head was pinned, followed by image guided registration. (E) The submucosal transseptal area was incised and (F) Mucosal flap was lateralised off the nasal septum. (G) Insertion of hardy speculum allows a better view of the sellar floor

For the microscopic technique (Figure 1), a single nostril approach was utilised, in which the submucosal transseptal area was identified and incised. The mucosal flap was lateralised off the nasal septum. This route will reach the junction of the nasoseptal bone and cartilage, and the area was torn to allow the insertion of a Hardy speculum. Then, the vomer was identified till the point of the anterior nasal spine and fractured. With the aid of image-guided surgery, the ostium and sphenoid sinus were located and enlarged. An ultrasonic Doppler probe was used to localise the internal carotid artery. The sella floor was breached with the use of a high-speed drill, exposing the dura and the opening was then enlarged with a Kerrison Rongeur and/or Stammberger punch. A dura incision was made in a cruciate manner. The tumour was removed with standard instruments such as ring curettes, dissectors, suction and micro forceps. A reconstruction of the sella defect was made with sandwich layers of oxidised cellulose plus gelatine sponge packing, overlaid with bony fragments and fibrin glue. The nasal mucosa was replaced with Vicryl 4/0 and nasal packing was inserted to support the sella base reconstruction.

**Figure 2:**
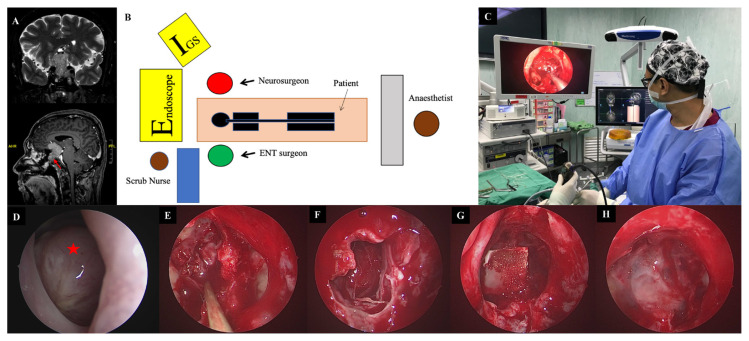
Illustration of endoscopic TSS. (A) A T2W (above) and T1 postgadolinium (below) MRI brain shows a sella and suprasellar lesion in a 45-year-old male who presented with worsening headache and bitemporal hemianopia. The tumour was seen extending into the sphenoid sinus (arrow). (B–C) Depictions of the OT arrangement, with the otorrhinolaryngologist and neurosurgeon standing by the patient. (D) Upon insertion of the endoscope, the tumour (star) was seen protruding through the sphenoid sinus. (E) The sphenoid phase with the visualised tumour. (F) The sellar phase after removal of the tumour with visualised diaphragmatic sella. (G–H) Sellar reconstruction was performed with a sandwich of oxidised cellulose plus gelatin sponge, layered with tissue glue

For the endoscopic TSS (Figure 2), both nostrils were utilised. The otorhinolaryngologist facilitated the pedicled nasoseptal step. Basically, the lateral wall of the nasal septum and the middle turbinate was infiltrated with marcain 0.5% and adrenaline 1:200,000 to achieve good decongestion. As previously described (12), the Hadad flap was performed. The most commonly used endoscope is a rigid endoscope 4 mm in diameter, 18 cm in length and with 0°, 30° and 45° lenses, according to the different steps of the surgical operation. The standard surgical techniques include three phases: i) nasal, ii) sphenoidal and iii) sellar. The first two phases were performed by the ENT team, while the sellar phase, including the tumour removal and sellar reconstruction, was performed by both the otorhinolaryngologist and the neurosurgeon. After the introduction of the endoscope into the nasal cavity, the middle turbinate was identified, pushed or dislocated laterally to widen the space between the turbinate and nasal septum. The inferior turbinate and roof of the choana were identified. Superiorly about 1.5 cm above the choana, posterior to the tail of superior turbinate and along the sphenoethmoidal recess, the sphenoid ostium was identified, to be followed by sphenoidotomy. During the sphenoid phase, the sphenoid ostium and part of the mucosa were removed to expose the sellar floor, planum sphenoidale, carotid prominence, opticocarotid recess and clivus, respectively. During the sellar phase, the sellar floor was removed, the dural matter was opened and the lesion was removed by means of gentle suction, a ring curette and a tissue grasper.

After tumour removal and haemostasis, the sellar reconstruction was performed using autologous or heterologous tissue grafts (fat, muscle, fascia or vascularised pedicle flap) or synthetic materials (oxidised cellulose, gelatin sponge or tissue glue) to fill the resection site and create a watertight closure.

### Statistical Analysis

The TSS cases were divided into two groups; i) microscopic and ii) endoscopic, to calculate the differences between the observed means in two independent samples. Data were expressed as mean ± standard deviation (SD) or median and (range) for continuous variables. Statistics of means, using Student’s *t*-test for unpaired data, was used to compare continuous variables between two groups. Statistics of categorical variables were calculated using a Chi-square and Fisher’s exact tests, as appropriate. A *P*-value (two-sided) less than 0.05 was considered to indicate statistical significance. All calculations were generated using GraphPad Prism 8.4.3 (GraphPad Software, Inc., San Diego, CA).

## Results

A total of 56 TSS were performed between August 2011 until June 2020 (Table 1). Thirty-seven patients (66.1%) were treated with microsurgical TSS and 19 (33.9%) with endoscopic TSS. There were no pre-operative differences between the endoscopic and microscopic groups, 31 of whom were men and 25 women; the male/female ratio was 1.24:1 (*P* = 0.780). The median age at surgery was 49 years old (range 14–70 years old; *P* = 0.860). Five patients in the endoscopic group (26.3%) had undergone prior transsphenoidal operations. There were no differences in terms of length of stay and intensive care unit stay between the group, with a median of 11 days and 2 days, respectively. More than half of patients that underwent microscopic group (*n* = 22 [59.5%]) were symptomatic with raised intracranial pressure symptoms, as compared to 36.8% in the endoscopic group (*P* = 0.161). Twenty-two patients (13 in the microscopic group (35.1%) and 9 in the endoscopic group (47.4%) were receiving suppressive medical therapy prior to hospital admission (*P* = 0.400), while the remaining were only started following pre-operative in hospital workup.

The histopathological diagnosis was examined as per standard histological examination of paraffin-embedded tissue sections and immunocytochemical studies. Non-functioning pituitary adenoma was the most common and confirmed in 30 patients (51.7%), with four of the patients (7.1%) presented with apoplexy. The pre-operative volume was nearly similar for the two groups (*P* = 0.930), however, there is a trend towards smaller post-operative volume in microscopic groups, with a total volume reduction of 79.0% (*n* = 36) as compared to the endoscopic group (*n* = 12 [63.0%]; *P* = 0.080). As confirmed by post-operative MRI, only 13 of 48 patients had complete tumour resection and there were no differences between the groups (11 of 36 patients (30.6%) in the microscopic group versus 2 of 12 patients (16.7%) in the endoscopic group; *P* = 0.500).

Next, intra-operatively, blood transfusions were needed for 9 of the 56 patients (16.1%); 6 of the 37 in the microsurgical group (16.2%) and 3 of the 19 in the endoscopic group (15.8%; *P* = 1.000) (Table 2). The indications were secondary to cavernous sinus injury or persistent mucosal bleeding. A total of 12 patients (21.4%) were documented to have CSF leaks, with seven of the patients requiring continuous lumbar drainage, while the other five patients were deemed satisfactory following primary closure. There were no differences between the groups (*P* = 0.730). During the first 5 years of implementing endoscopic TSS, three patients had an elective lumbar drain inserted prior to surgery. There was no documented CSF fistula post-operatively. For the operating time, there was a trend towards a longer duration for the endoscopic compared to the microscopic group (263 min versus 216 min; *P* = 0.080). The majority of the patients (67.9%) had tracheal extubation immediately after surgery and no differences were identified between the groups (*P* = 0.560).

**Figure 3:**
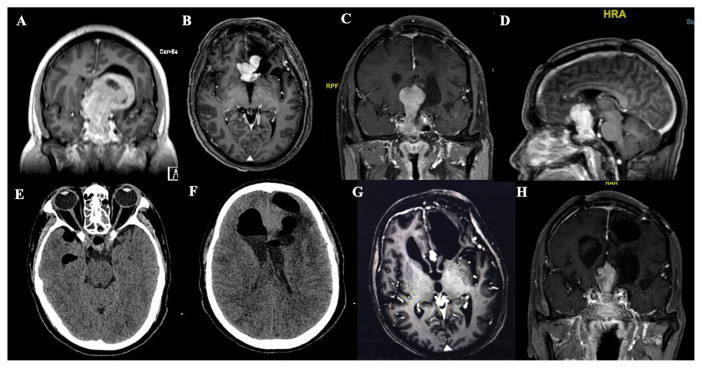
Case illustration. (A) T1W postgadolinium MRI brain of a 53-year-old male who was a known case of non-functioning pituitary macroadenoma. He underwent a tumour debulking via craniotomy in 2017. He consented to endoscopic TSS after MRI imaging showed a residual tumour, despite having no raised intracranial pressure (ICP) symptoms. (B–D) Pre-operative MRI brain with gadolinium, showing the tumour extending into anterior cranial fossa with widening of the sella turcica. (E–F) The patient developed post-operative CSF fistula, with the CT brain showing pneumocephalus. He subsequently underwent an EVD with transnasal repair of the defect. (G–H) MRI brain with gadolinium a year after surgery shows the residual tumour

The morbidity and mortality of TSS are summarised in Table 3. Post-operatively, 6 of the 56 patients (10.7%) were found to have CSF fistula. Four of the patients required placement of a lumbar drain. One patient from the microsurgical group required placement of an external ventricular drain (EVD). Another patient from the endoscopic group required an EVD and transnasal closure due to concomitant post-operative sella haematoma and pneumocephalus (Figure 3). No significant difference was found between the groups (*P* = 1.000). Similarly, for the sinus complications, there were no differences between the groups (*P* = 0.320), with an overall rate of 8.9%. The complications were only identified during outpatient visits and those who suffered from rhinosinusitis (*n* = 3 [5.4%]) required hospital admission and antibiotic treatment. Those that developed crusting and/ or polyps (*n* = 2 [3.6%]) required a release and/or polypectomy under local anaesthetic, without further sequelae. The risk of epistaxis was significantly higher in the endoscopic group (*n* = 4 [21.1%]) compared to the microscopic group, where none of the patients suffered the event (*P* = 0.010). Two patients developed epistaxis for the first 4 years of practice while another two patients in the subsequent 4 years.

For the endocrine outcome, the patients were categorised into transient and permanent, for both diabetes insipidus and anterior pituitary hormone deficiency. Overall, diabetes insipidus occurred in 19 of the 56 patients who underwent TSS (33.9%). Of the 13 patients with transient diabetes insipidus, eight underwent microscopic TSS (21.6%) and five underwent endoscopic TSS (26.3%; *P* = 0.730). Meanwhile, of the six patients with permanent diabetes insipidus, three underwent microscopic TSS (8.1%) and three underwent endoscopic TSS (15.8%; *P* = 0.380). For the anterior pituitary hormone deficiency, there were a total of 24 cases (42.9%). Upon review, 7 of the 37 patients (18.9%) in the microscopy group had transient hormone deficiency, while no such cases were identified from the endoscopy group (*P* = 0.080). New, permanent hormone deficiency occurred in 10 of the 37 (27%) patients in the microscopy group and 7 of the 19 (36.8%) in the endoscopy group. The incidence did not differ between the groups (*P* = 0.580).

The general complication rate was 35.7% (20 cases) and 65% (13 of the 20 cases) was from the endoscopic group. Respiratory events (eight patients, 14.2%) were the most frequently encountered in this category and was significant (*P* = 0.014). This was followed by central nervous system (CNS) events, occurring in four separate patients (7.1%); two patients had generalised tonic-clonic seizure necessitating reintubation, one patient had acute cerebral infarct and one patient had meningitis. One example was a 67-year-old lady who underwent endoscopic TSS. She was reintubated on post-operative day 1 due to type 1 respiratory failure. This was complicated with ventilator-associated pneumonia (VAP), acute cerebral infarct and sepsis. Due to her poor recovery, a tracheostomy was inserted but she later expired from general complications. The mortality rate was 8.9%, with two patients in the microscopic group and three in the endoscopic group (*P* = 0.300). Four patients expired due to deterioration in their medical conditions, particularly respiratory complications and septicaemia, while one patient expired from disease progression.

Table 4 shows the pre-operative and post-operative visual assessments. Pre-operatively, more than half of the patients presented with visual field, with and without visual acuity deficits (40 out of 56 patients [71.5%]). A visual assessment is not an indication of whether a patient should undergo microscopic or endoscopic TSS (*P* = 0.761). Post-operative assessment prior to discharge was available in 51 patients (35 in the microscopic group and 16 in the endoscopic group). This was compared with the baseline and categorised into ‘worse’, ‘no improvement’, ‘somewhat improved’, ‘resolved’ and ‘normal’. Three of the 16 patients from the endoscopic group had worse vision (18.8%; *P* = 0.032), while none from the microscopic group. Next, post-operative assessment during the 12-month visit was available in 50 patients (35 in the microscopic group and 15 in the endoscopic group) and compared to that prior to discharge. Of those three patients that had worse vision prior to discharge, two showed no improvement (similar to the post-operative findings) and one was shown to be somewhat improved (where the vision returned to the baseline). Overall, there were no differences between the groups in all categories.

## Discussion

The evolution of minimally invasive surgeries has been the catalyst for greater interest in endoscopic TSS. The growing attraction is due to the ability of the endoscope to enter the sella turcica and explore the lateral spaces in search of tumour remnants (13). Further refinements over recent decades have allowed better panoramic and/or angle views independent of the depth and width of the access routes (14). However, this is monocular vision. Hence, microscopic surgery, despite its relatively narrow field, gives true binocular three-dimensional vision, allowing precision during dissection (15). The desire of neurosurgeons for radical undertakings must be considered by weighing the risk and benefit of each technique. Variable results support better outcomes when performed via endoscopic (14, 16, 17) or microscopic (18) surgery. Other studies also reported comparable results from both techniques, regardless of the size or degree of invasion (19, 20).

In this institutional series, when the post-operative radiological findings were analysed, we found that the group treated via microscopic surgery had a higher percentage of tumour volume reduction than those who had undergone endoscopic surgery (79% versus 63%). Different results were noted to those produced by other institutions, which reported a higher percentage of total resection with endoscopic surgery, especially with tumour invading cavernous sinus (1), shorter operating time (10) and hospital stay (14). The possible reasons for this shortcoming are microscopic TSS has been longer in practice than endoscopic TSS and different combination of surgeons comprising both neurosurgeons and otorhinolaryngologist with variable depth of experience involved in endoscopic TSS at our centre.

CSF fistula remains a challenge to neurosurgeons. In the majority of cases, a CSF leak was detected intra-operatively and secured either through primary repair and/ or insertion of a lumbar drain. Post-operative CSF leak could still occur and may not be detected during surgery. Previous evaluation has already been conducted, supporting the view that there were no differences in terms of the rate of CSF leak, whether performed via endoscopic or microscopic surgery (21). In a high volume centre, the risk was similar with a general percentage of 2.6% (22). In terms of pre-operative lumbar drain placement, the dogma of elective insertion was to minimise the risk of post-operative CSF fistula and facilitate the healing of the dural repair under decreased tension (23). A recent study considered an elective lumbar drain had no added value and showed no differences in the event of post-operative CSF leak (24). At the start of endoscopic surgery, we practiced an elective lumbar drain. However, the approach was reverted to only when indicated. Should a CSF fistula be confirmed post-operatively, the lumbar drain insertion was combined with acetazolamide medication (for 7 days–10 days) and complete bed rest. The merit of prophylactic antibiotics was not justified in the above cases. In this series, out of the patients with CSF fistula, one had meningitis and was successfully treated with antibiotic therapy, and without clinical sequelae.

Post-operative epistaxis after TSS, potentially related to injury to the posterior nasal branch of the sphenopalatine artery, occurs in a minority of patients. The reported incidence is either similar in both groups (21) or has a higher tendency when endoscopic surgery is performed (19, 25). Persistent haemorrhage during surgery, such as from mucosal bleeding, will affect OT time (16). If not properly secured, patients may have epistaxis post-operatively. In this study, epistaxis only occurred in patients who underwent endoscopic TSS; hence, the four involved patients were reviewed. Two had intra-operative blood loss of more than 500 mL and post-operatively had persistent epistaxis requiring blood transfusions and/or tranexamic acid. The other two patients had minimal bleeding intra-operatively; however, post-operative computed tomography (CT) brain confirmed haematoma around the surgical bed with intraventricular haemorrhage. Both patients had prolonged hospital stays, were complicated with sepsis and soon expired.

Our study shows that post-operative general complications were higher in the endoscopic group than in the microscopic group, particularly for respiratory events. Of the eight patients with respiratory complications, four had ventilator-associated pneumonia and grew resistant organisms, either *Klebsiella pneumoniae* carbapenem-resistant enterobacteriaceae (CRE), *Pseudomonas aeruginosa* CRE or *Escherichia coli* extended spectrum beta-lactamases (ESBLs). This led to prolonged intubation, while three of them needed tracheostomy. Others did not report such higher respiratory complications (26). Pulmonary embolism and deep vein thrombosis are common occurrences (26, 27); nevertheless, none of these events occurred to the patients in this study. One study identified pulmonary complications as a result of prolonged intubation (28). We noted that three out of the six patients in the endoscopic group had overnight ventilation (patients were extubated the next day). Due to retrospective data analysis, there was unclear documentation of the indication. For the other three patients in the endoscopic group, one was reintubated due to type 1 respiratory failure, one remained intubated and eventually had a tracheostomy, and the last patient had hospital-acquired pneumonia. We incline to claim that a longer OT time, prolonged intubation and possibly the patient’s comorbidity (e.g. smoking and sleep apnoea) may contribute to post-operative complications. Furthermore, four of the five patients who expired in this study did so due to deterioration in their respiratory condition. Hence, it is believed that the determinant factor of morbidity is not only tumour pathology, but it may be influenced by the patient’s general condition and their pre- and post-operative care. In the future, it will be worthwhile correlating patient comorbidity with post-operative outcomes. We intend to optimise pre-operative workups to assist post-operative recovery.

A continual refreshment of neuroanatomy is necessary to improve surgical technique and avoid injuring neighbouring structures such as the optic nerve and chiasm, adenohypophysis, suprasellar cistern and vascular elements. The ramifications of TSS; despite the manoeuvring of the rigid endoscope or changing the magnification and illumination of the microscope, are greater appreciations of the optic nerve, chiasm and major arteries, which can only be seen via craniotomy. Nevertheless, dissection of the brain and related structure carries higher complication rates (29). When post-operative vision is jeopardised, the stipulation includes the devascularisation of the optic apparatus, post-operative haematoma, cerebral vasospasm, prolapse of the optic chiasm into an empty sella (30) and longer operative hours. This study identified those three patients in the endoscopic group that showed worse vision in the post-operative assessment and it is believed that the combination of events contributed to the deterioration of vision. Two patients had intra-operative haemorrhages, requiring blood transfusions and showed further complications with CSF leaks requiring CSF diversion. Post-operative CT brain showed the presence of suprasellar and intraventricular haemorrhage. Another patient had intra-operative and post-operative CSF leak necessitating a lumbar drain. Post-operative CT brain showed similar findings as to the other two patients. Those three patients also had a longer OT time than average.

## Limitations

We are aware of the limitations of this study. The retrospective series has potential drawbacks. Since all patients were treated at a single institution, their selection and referral bias may be a limiting factor. Inaccuracies in medical records, such as patient history, the duration of documented complications and illegible handwriting may affect data analysis. It is believed that the number of patients should be higher than was gathered for this paper; hence, more work should be done to improve data collection at our institution.

## Conclusion

In this series, endoscopic TSS is comparable to microscopic TSS but has a higher complication rate notably due to steep learning curve required to gain the expertise. The study identified significantly higher general complication rates, increased incidence of epistaxis and worse vision at post-operative assessments prior to discharge in the endoscopic group, compared to the microscopic group. In a university teaching hospital, we continue to improvise the adopted endoscopic approach and at the same time teaching it to residents.

## Figures and Tables

**Table 1:** Demographic characteristics of patients who underwent transsphenoidal surgery

Variable	Total cohort *n* (%)	Microsurgical *n* (%)	Endoscopic *n* (%)	Significance (*P*-value)
Sex				0.780
Male	31 (55.4)	21 (56.8)	10 (52.6)	
Female	25 (44.6)	16 (43.2)	9 (47.4)	
Age (years old)^d^	49 (range 14–70)	49 (range 16–70)	47 (range 14–67)	0.860
Previous surgery	5 (8.9)	No	5 (26.3)	
Length of stay (days)^d^	11 (range 4–34)	11 (range 4–34)	12.0 (range 6–28)	1.000
ICU stay (days)^d^	2 (range 0–20)	2 (range 1–12)	3.5 (range 0–20)	0.810
Raised ICP symptoms	29 (51.8)	22 (59.5)	7 (36.8)	0.160
Suppressive medical therapy	22 (39.3)	13 (35.1)	9 (47.4)	0.400
Lesion types				
NFPA	25 (44.6)	18 (48.6)	7 (36.8)	
NFPA with apoplexy	4 (7.1)	3 (8.1)	1 (5.3)	
Prolactinoma	7 (12.5)	4 (10.8)	3 (15.8)	
Prolactinoma with apoplexy	1 (1.8)	1 (2.7)	–	
ACTH secreting	6 (10.7)	4 (10.8)	2 (10.5)	
Growth hormone secreting	7 (12.5)	5 13.5)	2 (10.5)	
Craniopharyngioma	2 (3.6)	1 (2.7)	1 (5.3)	
Rathke’s cleft cyst	1 (1.8)	1 (2.7)	–	
Meningioma WHO grade I	2 (3.6)	–	2 (10.5)	
Fibrous dysplasia	1 (1.8)	–	1 (5.3)	
Pre-operative volume (cm^3^)^c^	8.2 (10)	11.0 (10)	12.0 (11)	0.930
Post-operative volume (cm^3^)^c^	3.7 (7.2)	3.2 (7.1)	5.2 (7.4)	0.120
Volume reduction (%)^a^, ^c^	75.0 (31)	79.0 (29)	63.0 (35)	0.080
Complete resection^b^	13 (27.0)	11 (30.6)	2 (16.7)	0.500

Notes: ACTH: adrenocorticotropic hormone; NFPA: non-functioning pituitary macroadenoma.

a,b There are eight missing values for variables ‘volume reduction (%)^a^’ and ‘complete resection^b^’. One missing in ‘microsurgical group’ and seven missing in ‘endoscopic group’;

c mean (SD);

d median (range)

**Table 2:** Intra-operative analysis of transsphenoidal surgery

Intra-operative variables	Total cohort *n* (%)	Microsurgical *n* (%)	Endoscopic *n* (%)	Significance (*P*-value)
Intra-operative transfusion	9 (16.1)	6 (16.2)	3 (15.8)	1.000
Intra-operative CSF leak				0.730
Lumbar drain	7 (12.5)	7 (18.9)	0	
No lumbar drain	5 (8.9)	2 (5.4)	3 (15.8)	
Total	12 (21.4)	9 (24.3)	3 (15.8)	
Elective lumbar drain	3 (5.4)	0	3 (15.8)	
Operating time (min)^a^	231 (72)	216 (60)	263 (85)	0.080
Timing of tracheal extubation				
Immediate	38 (67.9)	24 (64.9)	14 (73.7)	0.560
Overnight ventilation	18 (32.1)	13 (35.1)	5 (26.3)	

Note:

a mean (SD)

**Table 3:** Post-operative complications of transsphenoidal surgery

Post-operative complications	Total cohort *n* (%)	Microsurgical *n* (%)	Endoscopic *n* (%)	Significance (*P*-value)
CSF fistula				1.000
Lumbar drain	4 (7.1)	3 (8.1)	1 (5.3)	
EVD	1 (1.8)	1 (2.6)	0	
EVD and transnasal closure	1 (1.8)	0	1 (5.3)	
Total	6 (10.7)	4 (10.8)	2 (10.5)	
Sinus complications				0.320
Harvesting graft	0	0	0	
Rhinosinusitis	3 (5.4)	1 (2.6)	2 (10.5)	
Sphenoid polypectomy/septectomy	2 (3.6)	1 (2.6)	1 (5.3)	
Total	5 (8.9)	2 (5.4)	3 (15.8)	
Epistaxis				**0.010**
Observe	4 (7.1)	0	4 (21.1)	
Transnasal evacuation	0	0	0	
Diabetes insipidus^b^				
Transient	13 (23.2)	8 (21.6)	5 (26.3)	0.730
Permanent	6 (10.7)	3 (8.1)	3 (15.8)	0.380
Total	19 (33.9)	11 (29.7)	8 (42.1)	0.390
Anterior pituitary hormone deficiency^a^, ^b^				
Transient	7 (12.5)	7 (18.9)	0	0.080
Permanent	17 (30.4)	10 (27)	7 (36.8)	0.540
Total	24 (42.9)	17 (45.9)	7 (36.8)	0.580
General complications (cases) Respiratory event				**0.014**
Yes	8 (14.2)	2 (5.4)	6 (31.6)	
No	48 (85.7)	35 (94.6)	13 (68.4)	
Intravascular catheter related infection				0.339
Yes	1 (1.8)	0	1 (5.3)	
No	55 (98.2)	37 (100)	18 (94.7)	
Septicaemia				0.263
Yes	3 (5.4)	1 (2.6)	2 (10.5)	
No	53 (94.6)	36 (97.4)	17 (89.5)	
UTI				1.000
Yes	2 (3.6)	1 (2.6)	1 (5.3)	
No	54 (96.4)	36 (97.4)	18 (94.7)	
CNS event (e.g. seizure, cerebral infarction and/or meningitis)				0.591
Yes	4 (7.1)	2 (5.4)	2 (10.5)	
No	52 (92.9)	35 (94.6)	17 (89.5)	
Cardiac event				0.339
Yes	1 (1.8)	0	1 (5.3)	
No	55 (98.2)	37 (100)	18 (94.7)	
Exposure keratopathy				1.000
Yes	1 (1.8)	1 (2.6)	0	
No	55 (98.2)	36 (97.4)	19 (100)	
Total events	20	7 (18.9)	13 (65.0)	
Mortality	5 (8.9)	2 (5.4)	3 (15.8)	0.300

Notes: HAP: hospital acquired pneumonia; UTI: urinary tract infection;

a There is one missing value for variable ‘Anterior pituitary hormone deficiency’, that is in ‘Microsurgical group’;

b These variables are not mutually exclusive;

The highlighted *P*-value indicated significant result (< 0.05)

**Table 4:** Pre-operative and post-operative visual assessment

Visual assessment	Total cohort *n* (%)	Microsurgical *n* (%)	Endoscopic *n* (%)	Significance (*P*-value)
Pre-operative assessment				
Visual field deficits	17 (30.4)	13 (35.1)	4 (21.1)	
Visual acuity and field deficits	23 (41.1)	14 (37.8)	9 (47.4)	
Total	40 (71.5)	27 (72.9)	13 (68.5)	0.761
Post-operative assessment prior to discharge^a^, ^c^				
Worse	3 (5.9)	0	3 (18.8)	**0.032**
No improvement	7 (13.7)	5 (14.3)	2 (12.5)	1.000
Somewhat improved	25 (49.0)	18 (51.4)	7 (43.8)	0.760
Resolved	4 (7.8)	3 (8.6)	1 (6.3)	1.000
Normal	12 (23.5)	9 (25.7)	3 (18.8)	0.730
Post-operative assessment within 12-month^b^, ^c^				
Worse	1 (2.0)	0	1 (6.70)	0.300
No improvement	8 (16.0)	5 (14.3)	3 (20.0)	0.680
Somewhat improved	15 (30.0)	11 (31.4)	4 (26.7)	1.000
Resolved	17 (34.0)	13 (37.1)	4 (26.7)	0.530
Normal	9 (18.0)	6 (17.1)	3 (20.0)	1.000

Notes:

a There are five missing values for variables ‘Post-operative assessment prior to discharge’. Two missing in ‘Microsurgical group’ and three missing in ‘Endoscopic group’;

b There are six missing values for variables ‘Post-operative assessment within 12 months’. Two missing in ‘Microsurgical group’ and four missing in ‘Endoscopic group’;

c These variables are not mutually exclusive;

The highlighted *P*-value indicated significant result (< 0.05)
